# Evaluation of the Antinociceptive Action of Simvastatin in Mice

**DOI:** 10.7759/cureus.26910

**Published:** 2022-07-16

**Authors:** Nilesh T Katole, Jyoti S Kale, Harsh V Salankar

**Affiliations:** 1 Pharmacology and Therapeutics, Datta Meghe Medical College, Datta Meghe Institute of Medical Sciences, Nagpur, IND; 2 Physiology, Datta Meghe Medical College, Datta Meghe Institute of Medical Sciences, Nagpur, IND; 3 Pharmacology, Datta Meghe Medical College, Datta Meghe Institute of Medical Sciences, Nagpur, IND

**Keywords:** antinociception, simvastatin, writhing, statins, inflammatory pain, hot plate method

## Abstract

Introduction: Statins are well-established agents for dyslipidemia and have successfully been used for the prevention of coronary artery diseases for a long time; this is attributed not only to their lipid-lowering action but also to their pleiotropic actions. Recently many pleiotropic actions of statins have been explored, but very few studies were done to explore statins' antinociceptive action; therefore, the current study was planned to evaluate the antinociceptive activity of Simvastatin in different pain models in mice.

Materials and Methods: Antinociceptive activity of Simvastatin was evaluated by using Eddy's hot plate method (central analgesic model), acetic acid-induced writhing method (peripheral analgesic model), and biphasic formalin-induced paw licking method. Twenty-four mice were divided into four groups (n = 6 in each): Vehicle control group, simvastatin 5mg/kg, simvastatin 20mg/kg, and positive control group.

Results: In the hot plate method, as compared to the vehicle control group, Simvastatin 20mg/kg group showed a significant rise in the reaction time to the corresponding time interval (p<0.001). While the simvastatin 5mg/kg group did not show any significant analgesic activity in the hot plate test. In the acetic acid writhing method, both test groups show a significant delay in the onset of writhing and a decrease in the number of writhes as compared to the vehicle control group (P<0.001). While in the formalin test, both groups show dose-dependent analgesic activity in both the early and late phases.

Conclusion: Simvastatin exhibits analgesic activity in both central as well as peripheral models of analgesia, but central analgesia shows only at higher concentrations. Similarly, it inhibits inflammatory pain more predominantly than neurogenic, and hence simvastatin can be used in inflammatory conditions like rheumatoid arthritis and osteoarthritis particularly when there is coexisting dyslipidemia.

## Introduction

Pain is a protective mechanism. To relieve pain, the ideal method is to remove the cause of pain, but this is not always possible, feasible, and usually takes a long time; hence to get instant relief from pain, analgesics are used. Non-steroidal anti-inflammatory drugs (NSAID) and opioids are currently widely used as analgesics, but their use is limited by serious side effects [1]. Therefore, there is always a search going on for newer, safer, more effective analgesics, which is still not fulfilled.

Statins are the popular and well-recognized class of hypolipidemic agent that acts by inhibiting the “3-hydroxyl-3-methylglutaryl coenzyme A reductase” enzyme. Few recent studies have shown statin also poses pleiotropic effects like refining endothelial function, stabilization of atherosclerotic plaque, decreasing oxidative stress, inhibiting platelet aggregation, and anticoagulant effect [2-3]. There is also growing evidence from various studies that statins have additional anti-inflammatory and antinociceptive activity [4-5]. Few studies demonstrated that statins can prevent chronic inflammation in vivo [6-7].

Hence, it's very valuable to further explore these activities. There are studies available that show the antinociceptive activity of atorvastatin [8-11] and lovastatin [12,13], but very few studies are present which evaluated the antinociceptive activity of simvastatin in both central and peripheral models of analgesia. Therefore, the present study was planned to evaluate the antinociceptive activities of simvastatin in both models of central and peripheral analgesia.

## Materials and methods

Swiss albino mice of either gender weighing 20-30 grams were used. The mice were obtained from Haffkine Biopharma, Mumbai. Mice were kept in cages with a suitable air-cooling environment and provided 12 hours of light and dark cycles. They have free access to water and a standard laboratory diet. Mice were allowed to acclimatize to the conditions for seven days and were kept fasting overnight before the start of the experiment. This study was approved by the Institutional animal ethics committee (IAEC/DMIMS/2021/13), and the handling of animals was done as per CPCSEA (Committee for the Purpose of Control and Supervision of Experiments on Animals) guidelines.

Simvastatin was obtained in pure powdered form from Jubilant Organic Ltd, Mumbai. We tested simvastatin in 5mg/kg and 20mg/kg doses because it shows the minimum and maximum anti-inflammatory activity in reference studies [14]. Simvastatin was dissolved in 0.5% carboxymethylcellulose (CMC). Aspirin was used as a positive control in the acetic acid-induced writhing test method, while tramadol was used as a positive control for the hot plate method.

Four groups were made consisting of six mice each. Group 1: 0.5% CMC (vehicle control group) per oral, Group 2: Simvastatin 20 mg/kg per oral, Group 3: Simvastatin 5mg/kg per oral, Group 4: Positive control group-Tramadol 20mg/kg per oral (for hot plate method), Aspirin 100 mg/kg per oral (for writhing method and formalin method).

The Hot plate Method was performed as described by Vogel and Eddy et al. [15,16]. For this procedure, Eddy's hot plate was maintained at a constant temperature of 55 to 56⁰ Celsius. Each mouse was placed individually, and the time taken by the mice for either licking the paw or withdrawing the paws, or jumping off the surface, whichever was observed first, was taken as the endpoint. The reaction time was measured. To avoid damage to the paw, 15 second cut-off time was kept. Mean reaction time on the hot plate was noted at baseline and 20, 60, and 90 minutes after drug administration.

The writhing model of pain represents chemical nociception, and it is based on the principle that an irritant substance when injected into the peritoneum, it induced conditions similar to peritonitis, which produces writhes [15,17]. After a washout period of seven days, the mice were randomly assigned into four groups. Sixty minutes after receiving test drugs, 0.6% acetic acid of 0.1 ml was injected intraperitoneally into mice and placed in a clear glass chamber for observation of writhes. A writhing is marked by contractions of the abdomen with synchronous stretching of at least one hind limb. The number of writhes in 10 minutes, time of onset, and percentage inhibition were calculated based on the following formula.

Percentage inhibition = No. of writhing in the control group - No. of writhing in test group x 100/No. of writhing in the control group

In a formalin-induced paw licking test [15,18], mice were treated with a test drug. After one hour, each mouse injected 0.02 ml of 1% formalin subcutaneously in the right hind paw. The formalin injection produced a response in two distinct phases in mice. The first phase (0-5 min) indicates neurogenic type pain, while the second phase (15-30 min) indicates inflammatory type pain [18]. The time spent by mice in the licking paw was noted and expressed in % inhibition determined by the following formula.

Inhibition (%) = Paw Licking time in Control group - Paw licking time in test group x 100 /Licking time in Control group

No mice died during the experiment. Mice that belonged to the vehicle control group received standard analgesics after evaluation to decrease pain.

Statistical analysis: Data was analyzed by using IBM Corp. Released 2011. IBM SPSS Statistics for Windows, Version 20.0. Armonk, NY: IBM Corp. Comparison of intra-groups before and after the intervention was done by using paired t-test, while comparison between two groups was done by unpaired t-test. P-value <0.05 was considered to be statistically significant.

## Results

In the hot plate test (Table 1), the baseline reaction time in all the study groups is comparable (p>0.05). Simvastatin 20mg/kg group showed a significant increase in reaction time compared to the baseline and vehicle control group at 20-, 60-, 90-, and 120-minute intervals (P <0.001). The overall analgesic effect in the simvastatin20mg/kg group was observed from as early as 20 min post-dosing, and there was a gradual increase in reaction time till 120 minutes interval. These findings suggest early analgesic activity of simvastatin at a 20 mg/kg dose, which continued till the duration of the evaluation. However, no significant increase in reaction time was observed in simvastatin 5mg/kg. (p> 0.05). Reaction times in the tramadol group at 20, 60, 90, and 120 minutes were significantly more compared to that in the vehicle control group as well as the simvastatin 5mg/kg and 20mg/kg group evaluated at the same periods (P<0.0001).

**Table 1:** Effect of different drugs on nociception in Hot plate method of analgesia in mice. Values are mean ± SD, n = 6 in each group, p.o.: per oral. * P<0.001 when compared to its basal reaction time. † P<0.001 when compared to 0.5% CMC group. ‡ P<0.001 when simvastatin 20mg/kg group compared to Tramadol group.

Groups	Basal reaction time (in seconds)	20 min interval (in seconds)	60 min interval (in seconds)	90 min interval (in seconds)	120 min interval (in seconds)
0.5% CMC	3.8±0.59	3.68±0.68	3.62±0.99	3.9±0.94	3.62±0.33
Simvastatin 5mg/kg p.o	3.6±0.75	4.1±0.56	3.9±0.15	4.3±0.78	3.9±0.4
Simvastatin 20mg/kg p.o	3.2±0.5	5.3±0.72^*^^†‡^	7.16±0.64^*^^†‡^	9.3±0.45^*^^†‡^	10.2±0.33^*^^†‡^
Tramadol p.o.	3.3±0.73	8.5±0.13^*^^†^	11.8±0.47^*^^†^	17.5±0.55^*^^†^	18.2±0.16^*^^†^

In the writhing method (Table 2), the in-vehicle control group onset of writhing was very quick (269 ± 29.4 seconds), and the average writhes in 10 minutes was also highest (29.83± 1.94) compared to other groups. The aspirin group showed a significant delay in the onset of writhing (753± 61.4 seconds, p<0.001) and also showed a significant decrease (p<0.001) in the average number of writhes to the lowest (8.83±1.16). The percentage inhibition with aspirin was highest (70.39%). The simvastatin 20mg/kg group showed a significant delay (p<0.001) in the onset of writhing (542 ±37.8 seconds) and also showed a significant decrease (p<0.001) in the number of writhes in 10 minutes (15.33±1.21) as compared to the vehicle control while the percentage of inhibition was 48.60%. The simvastatin 5mg/kg group also showed a significant delay (p<0.001) in the onset of writhing (343±26.3 seconds), and the number of writhes in 10 minutes also decreased (24 ± 1.7). The percentage of inhibition was 19.69%.

**Table 2:** Effect of different drugs on acetic acid induced writhing method. Values are mean± SD, n=6 in each group, *P<0.001 when compared to 0.5% CMC group. †p<0.001 when compared to the Aspirin group.

Groups	Time of onset (seconds)	Number of writhes in 10 minutes	Percentages inhibition
0.5% CMC	269 ± 29.4	29.83± 1.94	----
Aspirin 100 mg/kg group	753± 61.4*	8.83±1.16*	70.39%
Simvastatin 20mg/ kg group	542 ±37.8*^†^	15.33±1.21*^†^	48.60%^†^
Simvastatin 5mg/kg group	343±26.3*^†^	24±1.41*^†^	19.69%^†^

Formalin-induced paw licking: (Table 3), the vehicle control group showed the highest licking time in both phases (91.14 seconds and 110.83 seconds, respectively). The aspirin group showed a significant reduction in licking time in both the early phase (48.8 sec) and late phase (23.24 sec) as compared to the vehicle control group 91.14 sec and 110.8 sec, respectively (p<0.001). The percentage inhibition was 46.45% in the early phase and 78.87% in the late phase. The simvastatin 20mg/kg group showed a significant reduction in paw licking time in both the early phase (59.95 sec) and late phase (38 sec) as compared to the vehicle control group (P<0.001). The percentage inhibition was 34.06% in the early phase and 65.45% in the late phase. Thus, simvastatin 20mg/kg showed more protection from inflammatory pain than neurogenic pain. The simvastatin 5mg/kg group also showed a significant reduction in licking time in the early phase 87.23 sec and 68.14 sec in the late phase (P<0.001) as compared to the vehicle control group. The percentage inhibition was 4.29% in the early phase and 38.18% the late phase.

**Table 3:** Effect of different drugs on formalin paw licking test of analgesia in mice. Values are mean ± SD, n = 6 in each group. *P<0.001 when compared to 0.5% CMC group. †p<0.001 when compared with the Aspirin group.

Groups	Early phase		Late phase	
	Paw Licking time (seconds)	Percentage inhibition	Paw Licking time (seconds)	Percentage inhibition
0.5% CMC	91.14±1.72	-------	110.83±1.93	-----
Simvastatin 5mg/kg	87.23±1.5*	4.29%^†^	68.14±1.72*†	38.18%^†^
Simvastatin 20mg/kg	59.95±1.76*^†^	34.22%^†^	38±1.78*^†^	65.45%^†^
Aspirin 100mg/kg	48.8±1.69*	46.45%	23.24±1.36*	78.87%

## Discussion

Statins have been widely recommended in clinical guidelines as frontline drugs to treat dyslipidemia and prevent cardiovascular disease for a long time. More recently, there has been growing interest in its pleiotropic and non-hypolipidemic actions. There are reports which show statin's antinociceptive and anti-inflammatory activities. Among all statins, simvastatin is a strong lipophilic that can enter the brain and, therefore can alter central pathways of pain.

We evaluated antinociceptive activity using three models of pain. The hot plate method is an effective and sensitive model to evaluate the efficacy of centrally-acting analgesic drugs. Therefore, tramadol showed maximum pain threshold while Simvastatin shows central analgesic activity only at the higher dosage. Similar findings were seen in previous studies on atorvastatin and lovastatin by hot plate method. In some studies, statins did not show any significant activity in the hot plate method [19], while in other studies, analgesic activity was seen only at higher doses [9]. The second method was the acetic acid writhing test which represents the chemical nociception model; it’s a sensitive test to evaluate peripheral analgesics pathways. In this method, simvastatin 20mg/kg and 5mg/kg groups showed a delay in the onset of writhing and a decrease in the number of writhes as compared to the vehicle control group. Thus, both groups showed peripheral analgesia. The results obtained in the writhing test are in conjunction with previous works in which other statins like atorvastatin and lovastatin were evaluated using the writhing test [9,19]. The third method was the biphasic Formalin test which was used to discriminate between neurogenic pains from inflammatory pain both the test group simvastatin 20mg/kg and simvastatin 5mg/kg showed protection against formalin-induced paw pain, though the effect was minimal in the latter group. This finding suggests that simvastatin has dose-dependent analgesic activity in both neurogenic pain and inflammatory pain. The results of the present study reveal consistent antinociception activity of simvastatin in different animal models. This proves that statins have an antinociceptive property, and they act by both central and peripheral mechanisms; it has more activity against inflammatory pain than neurogenic pain.

In our study, simvastatin’s mechanism of action regarding antinociception is not studied, but the results of other authors suggest that it might involve the inhibition of various mediators and cytokines involved in inflammatory hypernociception.

T Santodomingo-Garzon et al. [10] have reported that atorvastatin reduced hypernociception by reducing bradykinin, tumor necrosis factor-α, IL-1b, and chemokine CXCL factors, while in another study simvastatin was found to inhibit prostaglandin E2 which suggest simvastatin's suggesting direct action on peripheral nociceptors. Supporting this assumption, some studies also claimed that the anti-inflammatory action of atorvastatin is comparable to the diclofenac [11]. Hernández-Presa MA et al. demonstrated that statins can reduce cyclooxygenase-2 (COX-2) protein expression; therefore, the antinociceptive activity of statins can be due to the inhibition of cyclooxygenase-2 expression [8]. Several studies also showed that statins increase the bioavailability of Nitric oxide (NOS) by upregulating NOS, which can be one of the reasons for its antinociceptive action [20,21].

Some studies proposed that inhibition of nuclear factor kappa B by statins leads to inhibition of release of prostaglandin and cytokines, which can be one more reason for its antinociception action [22,23].

Limitation of the study

Further evaluation of the simvastatin-like assessment of cytokine levels, e.g., bradykinin, tumor necrosis factor-α, IL-1b, and COX 2 protein expression, will precisely explore the exact mechanism of antinociceptive action of simvastatin, which is a major limitation of this study.

## Conclusions

In this study, we evaluated the antinociceptive activity of simvastatin in both central and peripheral animal models. We found that simvastatin inhibits both central and peripheral pain in a dose-dependent manner, and it inhibits inflammatory nociception predominantly than neurogenic pain. In this study, we reaffirm that statins could be a novel class of analgesic agents. As statins have good analgesic activity predominantly, inflammatory type, they can be used for chronic inflammatory conditions like rheumatoid arthritis and osteoarthritis, particularly when there is coexisting dyslipidemia. Statins could be used as an adjuvant with other analgesic drugs in both acute and chronic painful conditions.
